# Study protocol of effectiveness of a biopsychosocial multidisciplinary intervention in the evolution of non-speficic sub-acute low back pain in the working population: cluster randomised trial

**DOI:** 10.1186/1472-6963-10-12

**Published:** 2010-01-12

**Authors:** Teresa Rodriguez-Blanco, Isabel Fernández-San-Martin, Montserrat Balagué-Corbella, Anna Berenguera, Jenny Moix, Elena Montiel-Morillo, Esther Núñez-Juárez, Maria J González-Moneo, Magda Pie-Oncins, Raquel Martín-Peñacoba, Mercè Roura-Olivan, Montse Núñez-Juárez, Enriqueta Pujol-Ribera

**Affiliations:** 1 Institut d'Investigació en Atenció Primària Jordi Gol (IDIAP Jordi Gol), Institut Català de la Salut, C/Gran Via de les Corts Catalanes 587 àtic, 08007 Barcelona, Spain; 2 SAP Litoral, Institut Català de la Salut, C/Lope de Vega 138, 08005, Barcelona, Spain; 3 Societat Catalana de Medicina Familiar i Comunitària (CAMFIC), C/Portaferrissa n°8 Pral, 08002 Barcelona, Spain; 4 Facultat de Psicologia, Universitat Autònoma de Barcelona, 08193 Bellaterra, Barcelona, Spain; 5 Hospital de Sant Pau, C/Sant Antoni Maria Claret 167,08025 Barcelona, Spain; 6 SAP de suport al Diagnostic i Tractament, Institut Català de la Salut, Av. De les Drassanes, 17-21, 08001 Barcelona, Spain; 7 EAP Verneda Nord, Institut Català de la Salut, Pl. de la Infància, s/n, 08020, Barcelona, Spain; 8 EAP Manso, Institut Català de la Salut, Manso 19, 08015 Barcelona, Spain; 9 Hospital Clínic i Provincial de Barcelona, Villarroel 170, 08036, Barcelona, Spain

## Abstract

**Background:**

Non-specific low back pain is a common cause for consultation with the general practitioner, generating increased health and social costs. This study will analyse the effectiveness of a multidisciplinary intervention to reduce disability, severity of pain, anxiety and depression, to improve quality of life and to reduce the incidence of chronic low back pain in the working population with non-specific low back pain, compared to usual clinical care.

**Methods/Design:**

A Cluster randomised clinical trial will be conducted in 38 Primary Health Care Centres located in Barcelona, Spain and its surrounding areas. The centres are randomly allocated to the multidisciplinary intervention or to usual clinical care. Patients between 18 and 65 years old (n = 932; 466 per arm) and with a diagnostic of a non-specific sub-acute low back pain are included. Patients in the intervention group are receiving the recommendations of clinical practice guidelines, in addition to a biopsychosocial multidisciplinary intervention consisting of group educational sessions lasting a total of 10 hours. The main outcome is change in the score in the Roland Morris disability questionnaire at three months after onset of pain. Other outcomes are severity of pain, quality of life, duration of current non-specific low back pain episode, work sick leave and duration, Fear Avoidance Beliefs and Goldberg Questionnaires. Outcomes will be assessed at baseline, 3, 6 and 12 months. Analysis will be by intention to treat. The intervention effect will be assessed through the standard error of measurement and the effect-size. Responsiveness of each scale will be evaluated by standardised response mean and receiver-operating characteristic method. Recovery according to the patient will be used as an external criterion. A multilevel regression will be performed on repeated measures. The time until the current episode of low back pain takes to subside will be analysed by Cox regression.

**Discussion:**

We hope to provide evidence of the effectiveness of the proposed biopsychosocial multidisciplinary intervention in avoiding the chronification of low back pain, and to reduce the duration of non-specific low back pain episodes. If the intervention is effective, it could be applied to Primary Health Care Centres.

**Trial Registration:**

ISRCTN21392091

## Background

Low back pain (LBP), in any of its forms (acute, sub-acute or chronic), is a common and important reason for consultation with the general practitioner (GP). The point prevalence rate of LBP has been estimated as 14.8% in a study carried out in the general Spanish population [1], while the same study finds a probability of 44.8% of presenting at least one episode of LBP in a period of six months, similar to the annual prevalence of 41% observed in the Dutch population [2,3].

LBP has an important impact on the performance of daily tasks in individuals who present the condition and on their quality of life. It also has family and social repercussions, which are often ignored [1,3]. It is one of the most frequent causes of absenteeism, estimated at an average of 21.9 days of work sick leave per episode [4]. However, most data related to daily activity come from the field of work and exclude relevant groups such as housewives; thus the consequences of this problem are clearly underestimated [1].

A study carried out in 36 Primary Health Care Centres (PHCC) in Spain describes the management of a patient with non-specific LBP. Courses of action taken include: 98% of the patients receive pharmacological treatment, 19% some kind of physical therapy and 10% are referred to surgery, while 43% of the patients are assessed through radiological techniques. This study showed that, although management of the patients is consistent with the recommendations based on evidence, after two months of treatment, the pain continued in 37% and had worsened in 10% of patients [5].

Multi-causality of LBP limits, in some cases, the election of an approach strategy which improves symptomatology and obtains effective results. Little is known about effectiveness of primary prevention, both in the general population and in the work place. A systematic revision undertaken by Linton et al [6], observed that physical exercise alone showed a weak, though consistent, preventive effect. However, the US Preventive Services Task Force [7] does not find sufficient evidence to support the recommendation for or against postural exercises or modification of risk factors to prevent LBP.

Negative findings obtained in many preventive studies might be explained by various factors. For instance, some studies present methodological defects (small samples, short following-up periods, compliance not always high). Moreover, included populations represent a high variety of occupations, and interventions are too variable in content, frequency and duration [6].

When the problem has already been established, early interventions are necessary to prevent chronicity. Selection procedures of psychosocial risk factors have been used to identify patients with a higher risk of developing long-term disability. In those patients, a cognitive-behavioural interventional approach has proved effective in diminishing that risk [8,9]. Therefore, any LBP approach should consider factors associated with the symptom, which not only can affect the degree of pain, but can increase the risk of it becoming chronic, as in the case of patients with psychological [3,10,11] and/or social aspects.

There is evidence of effectiveness of intensive biopsychosocial multidisciplinary intervention in patients with non-specific chronic LBP, according to a systematic review of randomised controlled trials [12].

A Cochrane review, which assesses the same objective but in sub-acute LBP in adults, only includes two randomised clinical studies which meet criteria for sub-acute LBP (between 4 weeks and 3 months duration), and both studies are classified as being of a low methodological quality. The authors conclude that studies with a high methodological quality are necessary, which include bigger sample size and adequate duration of follow-up to test the objectives[13].

It is generally accepted that LBP becomes chronic when the pain persists for longer than 3 months or occurs episodically within a 6 month period [3,14]. Sub-acute LBP occurs suddenly after a period of at least 6 months without LBP and there is a variability of the duration criteria, which range from 2 to 6 weeks. Research conducted in Spain indicates that after 2 weeks of pain, changes occur in the factors which affect disability and quality of life [15]. The same study associates disability and low quality of life perception with the duration of pain, more than with the degree of pain.

The introduction of clinical practice guidelines in the treatment of LBP [16,17] has incorporated diagnostic aspects, effectiveness of the pharmacological and non-pharmacological resources and patient educational level. However, it may be necessary to integrate other multidisciplinary treatment strategies (physical, psychological and social/occupational) which applied to patients with non-specific sub-acute LBP at the correct time, may avoid chronification of the condition, as well as reduce the individual social and economic impact which this condition has on society.

The main objective is to analyse the effectiveness of a biopsychosocial multidisciplinary intervention approach (rehabilitation or physiotherapy, cognitive-behavioural and pharmacological therapy), to improve disability, reduce pain severity, improve quality of life and reduce incidence of chronic LBP in the working population with non-specific LBP, compared to the usual clinical care.

The secondary objectives are:

- To assess differences between both groups of patients, the group receiving biopsychosocial multidisciplinary intervention and the group receiving usual clinical care, in terms of duration in days of the LBP episode, work sick leave, duration in days of the period of sick leave and pharmacological treatment.

- To assess the responsiveness of the Spanish version of the following questionnaires: Roland Morris disability (RDQ), Mc Gill Pain, Quality of Life (SF-12), Fear Avoidance Beliefs (FAB) and Goldberg questionnaires.

- To compare patient satisfaction with care between both intervention arms.

- To identify factors associated with chronification of a non-specific LBP episode: sociodemographic, clinical (duration of the current episode, severity and others) and psychosocial signs of bad prognosis **(**wrong beliefs, inadequate behaviour, working factors, mood).

## Methods/Design

### Study design

A cluster randomised controlled clinical trial will be carried out, analyst blinded, which compares patients with non-specific LBP treated with a multidisciplinary approach, with a control group receiving only usual clinical care.

### Setting

The trial will be conducted in a primary care setting, PHCCs located in Barcelona, Spain and its surroundings. We will contact all the PHCCs by presenting the study to their staffs and we will invite them to participate. The study will be comprised of at least 38 PHCCs.

### Study population

Eligible patients will be consecutively selected by their GP, who will inform them about the study objectives. All patients who agree to participate will be given written informed consent to sign.

Patients will be included if the current episode of LBP occurs suddenly after a period of a minimum of 6 months without LBP and lasts between 15 days and 12 weeks [15] and if they do not fulfill any of the exclusion criteria [3].

Furthermore, patients have to be between 18 and 65 years old, and understand Catalan or Spanish and will be accessible for at least twelve months.

Patients will be excluded if: they are unwilling to participate in the multidisciplinary intervention trial; LBP coexists with cognitive impairment or severe psychiatric disorders such as psychosis, or if they have severe major depression; any other cause of disability which impedes answering the various questionnaires; they are pregnant or breast-feeding; they might have anti-inflammatory intolerance or allergy; treatment for physical problems in the preceding three months has been given; and they have been referred for intensive functional restoration programmes or if they have a confirmed diagnosis of fibromyalgia.

Furthermore, the GP has to ensure that the patient has no red flag signs or symptoms that are frequently associated with specific LBP or potentially severe illnesses [5,16,17].

Red flags for possible specific spinal pathology are: oncologic disease during the previous 5 years; constitutional symptoms, unexplained weight loss, fever, chills, recent urinary tract infection; history of drug or alcohol abuse, or immunocompromised host; vertebral fracture (previous history of major trauma: traffic accidents or falls from heights: patients with risk factors of osteoporosis, long term treatment with glucocorticoids); inflammatory spondylarthropathy (SpA) (pain of typical inflammatory night-pattern pain which forces the patient to get up from bed, gradual onset of pain before the age of 40, severe morning stiffness, limited low back mobility on the sagittal/lateral plane, possible inflammatory involvement of peripheral joints, family history, extra-articular manifestations: uveitis, iritis, psoriasis, colitis, urethritis).

Red flags for surgical referral: saddle anaesthesia, recent onset of bladder dysfunction or anal sphincter impairment, major or progressive motor weakness, sensory level, or widespread neurological signs.

### Randomisation

In this study a cluster design is used because the intervention is delivered to groups. To minimise contamination, the unit of randomisation will be the PHCC. Those PHCCs who agree to participate will be randomly allocated to control or intervention groups by a random sequence generated by a computer programme in blocks of random size and prepared before recruitment of the PHCC by an independent statistician who will be blind to the PHCCs' identities. The blocking factor was randomly selected between an even number (e.g., 4, 6, or 8) and will vary as the recruitment continues. Primary care physicians will be informed about their allocation after giving final consent to participation.

To minimise imbalance across intervention groups, randomisation was conducted stratifying by percentage of immigrants from developing countries registered in each district. We consider two strata, less than and more than 15% immigrants. This variable is taken as a proxy of socioeconomic level.

### Blinding

During the recruitment, the GP will identify new episodes of sub-acute LBP in patients consulting for this reason and those who meet the inclusion criteria will be allocated to the intervention group corresponding to the centre. To avoid bias, consent to participate will be obtained before the allocation.

Because of the nature of the intervention, GPs were not blind to the patients' allocations. Data analysis will be carried out so that the intervention groups allocated to the patients will be unknown to the analyst.

### Intervention Design

Patients allocated to the control group will receive usual clinical care, and individual intervention based on the application of the "Clinical Practice Guidelines in the Pathology of the Lumbar Spine in Adults" recommendations, published by the "Institut Català de la Salut" [16]. Topics covered are explained in Table 1.

**Table 1:** Contents of the clinical guidelines applied in this cluster randomised trial

Clinical Practice Guidelines in the Pathology of the Lumbar Spine in Adults
▪ Patient education, give reassuring and positive information about the benign nature of LBP, offer written information including specific advice.
▪ Advise avoiding bed-rest and encourage the person to be physically active and continue with normal activities as far as possible.
▪ Consider offering a structured physical exercise program tailored to personal preferences
▪ Physical exercise should be introduced gently at first (walking, cycling, and swimming) and progressively increased in intensity.
▪ Recommend attendance at the "Back School" to those patients who have not resumed their daily tasks, after six weeks.
▪ Prescribe pharmacological treatment according to the established guidelines

Notes: LBP = Low back pain

Patients allocated to the intervention group will receive the same individual intervention described for the control group, in addition to an educational booklet "The Back Manual [18]" (transculturally adapted Spanish version of the Back Book [19]) and a biopsychosocial multidisciplinary group intervention.

This group intervention will be carried out by a GP, a nurse, a psychologist and a physiotherapist. The programme consists of 2 sessions of 4 hour duration each and 1 session of 2 hour duration. Each group includes up to 12 participants. Patients will be given a leaflet with detailed information of the contents of each session accompanied by examples and exercises, a compact disc of Jacobson's progressive relaxation technique and an audiovisual. The audiovisual reinforces the educational content of the group sessions, such as postural education and motivational and psychological aspects (thoughts on life values and the organization of time). Details of the biopsychosocial multidisciplinary group intervention and the educational digital video disc (DVD) are included in Tables 2 and 3.

**Table 2:** Components of the biopsychosocial multidisciplinary group intervention

GP + Nurse 2 hours	Objective: Resolve doubts, demystify concepts about LBP and promote adherence to the intervention
Theory program	▪ Basics on anatomy and biomechanics of the spine
	▪ Pain mechanisms
	▪ Causes of pain and predisposing factors
	▪ Type of pain, mechanical, inflammatory, and severity
	▪ Healthy life habits

Practical program	▪ Discuss with the participants the doubts, beliefs and myths about back pain and give positive messages

**Physiotherapist 4 hours**	**Objective: Provide tools on exercises/postures to avoid the pain and the chronic course and improve quality of life.**
Theory program	▪ Body posture and its implication in pain
	▪ Ergonomics
	▪ Benefits of relative rest

Practical program	▪ Diaphragmatic breathing exercises as the basis for relaxation, body awareness and postural control.
	▪ Pelvic floor/gyration exercises.
	▪ Propioceptive and posture awareness exercises.
	▪ Strengthening exercises of the psoas and the posterior chain: Paravertebral muscles, gluteus, ischiotibial muscles.
	▪ Strengthening exercises of abdominal muscles, specially the abdominal transversus, gluteus, spinal extensors and scapular muscles.

**Psychologist 4 hours**	**Objective: Provide participants with cognitive-behavioural therapy techniques**
Theory program	▪ Influences of cognitions, emotions and behaviour in pain

Practical program	▪ Relaxation guidelines and methods
	▪ Cognitive restructuring (Modulation of negative thoughts affecting emotions and pain)
	▪ Use of attention (Increasing attention focus)
	▪ Assertiveness (improving social relationships)
	▪ Problem solving (training in step by step techniques for decision making)
	▪ Time organization and reinforcement of reform activities and physical exercise.
	▪ Life values (increasing concordance between values and behaviour)
	▪ Relapse prevention

Notes: GP = general practitioner; LBP = Low back pain

**Table 3:** Contents of the educational Digital Video Disc

Basics on anatomy and biomechanics of the spine
Causes and mechanisms of pain
Recommendations on dealing with pain and coping with it in daily life
Ergonomics applied to daily life (home, work and leisure)
A series of stretching, strengthening, and flexibility exercises and methods to promote physical activity
Cognitive restructuring (Modulation of negative thoughts affecting emotions and pain)
Use of attention (increasing attention focus)
Assertiveness (Improving social relationships)
Problem solving (training in step by step techniques for decision making)
Time organization and reinforcement of reform activities and physical exercise
Life values (Increasing concordance between values and behaviour)
Relapse prevention

To guarantee the standardisation of the group sessions, only one qualified psychologist and one physiotherapist, both of them with extensive expertise in development of training groups, will apply the intervention in all PHCCs.

### Other non-pharmacological therapeutic measures and Patient Compliance

During the intervention period, other non-pharmacological therapeutic measures which differ from those stated in the intervention design will be advised against. However, in the case of non-pharmacological therapeutic measures occurring, they will be registered and included in the analysis.

In the follow-up visits at 3, 6 and 12 months, we will ask about the patient compliance of recommendations of treatment and the questionnaires will be completed. Furthermore, if the patient is unable to attend the follow-up visits, the data collection will be carried out by telephone call.

Compliance to the group sessions will be assessed by registering the number of group sessions that patients attend.

To ensure patients' adherence to the group sessions, they will be asked a time preference (morning or afternoon) to attend the sessions. One week prior to the group session, they will receive a phone call to report the date, time and location of the session and on the day before, an SMS message with the same information will be sent.

At the end of the study, the control group will receive the compact disc of Jacobson's progressive relaxation technique, the educational booklet "The Back Manual" and the audiovisual.

### Outcomes

Primary outcomes are:

- *Change in disability measured by Roland Morris disability questionnaire (RDQ) *[20,21]. The primary outcome is change in the score on the Roland and Morris disability questionnaire at three months after onset of pain.

Our study will use the Spanish version of the questionnaire, trans-culturally adapted and validated by the Kovacs Foundation, in collaboration with a team of researchers from various hospitals, including the Kovacs Foundation and different PHCCs. Conducted studies demonstrate the *RDQ *questionnaire is a reliable and sensitive measure to evaluate the disability which LBP may generate, namely the limitations in daily tasks which it may entail. Furthermore, the RDQ questionnaire may perform better in the general population [22]. Total scores are obtained through addition of scores for each selected statement, rating from 0 to 24.

- *Intensity of Pain assessed by Pain Questionnaire Spanish version (Mc Gill Pain Questionnaire, (Melzac, 1975) *[23-27], This is a self-administered questionnaire. It is based on the view of pain perception as multidimensional: sensory-discriminative, motivational-affective and cognitive-evaluative. It rapidly identifies the predominant components of pain perceived by the patients. It covers 62 descriptors distributed along 15 classes and 3 dimensions (sensorial, affective and evaluative). It assesses 3 parameters: Total Intensity score, Current Intensity score and Visual Analogical Scale (VAS). Total Intensity score is obtained by adding each class score. Each class score is obtained from ticking one or none of the descriptors in each class. If one is ticked, it scores 1, and if none are ticked, it scores 0. Total Intensity score, which will be located between 0-14, is obtained by adding these three dimensions. Current Intensity score is obtained from a scale such as Likert, rating from 0 to 5. Analogical Visual Scale rates from 0 to 10.

- *Quality of Life measured by SF-12*. We will use the Spanish version of SF-12 because for large group studies, the differences in measurement reliability between the SF-12 and SF-36 are not as important and the SF-12 is worthwhile for specific populations based on large sample sizes [28]. The 12 items provide a representative sampling of the content of the 8 health concepts and operational definitions of those concepts, including what respondents are able to do, how they feel, and how they evaluate their health status. The dimensions were physical function (2 items), physical role (2), social functioning (1), emotional health (2), mental health (2), vitality (1), bodily pain (1), general health (1). The answer options on the Likert scale evaluate the intensity and frequency of these dimensions. Time reference in the version used is 4 weeks. Scores rate from 0 for worse health status to 100 for best health status, for each of the dimensions. The questionnaire should preferably be self-administered.

Secondary outcomes are:

- *Duration of the current episode of LBP (pre-study and study duration)*. We will consider the current episode finished when the pain and its impact on the person's daily life is reduced, even when the pain cannot be cured completely [29].

- *Work sick leave (yes or no)*

- *Duration in days of work sick leave*

- *Percentage of change in pharmacological treatments*.

- *Wrong beliefs, inadequate behaviour and work factors assessed by Fear Avoidance Beliefs Questionnaire (FAB) *[30]. Among psychosocial factors associated with LBP, avoidance behaviour of physical activity or work due to fear of pain have been demonstrated to have an influence on the disability. The Spanish version of the FAB questionnaire allows us to quantify beliefs and fears regarding the causes and consequences of pain, with validity and accuracy in patients with LBP. This questionnaire, recently validated, contains 16 statements (five related to physical activity and eleven to work activity), which patients score from 0 to 6 depending on their degree of agreement or disagreement. Thus, higher scores indicate a higher degree of avoidance behaviour. Completion takes around ten minutes and it can be self-administered.

- *Anxiety and Depression measured by The Spanish version of the Goldberg questionnaire, the "Escala Ansiedad y Depresión de Goldberg" (E.A.D.G) *[31]. This was designed to aid GPs and other non-psychiatrists in the better recognition of mental illness. Its validity and discriminatory power for detecting anxiety and depression has been demonstrated, as well as its usefulness as an interview guideline. Each scale has 9 items. The anxiety scale has 4 core symptoms (keyed up, worrying a lot, irritability and difficulty relaxing) and 5 supplementary symptoms (insomnia, headaches, autonomic symptoms, health worries and delayed sleep). The depression scale has 4 core symptoms (low energy, loss of interest, loss of confidence and hopelessness) and supplementary symptoms (inefficient thinking, poor appetite/weight loss, early waking, feeling sluggish and feeling worse in the morning). The interpretation is made by adding the anxiety score to the depression score. Patients with anxiety scores of five or depression scores of two have a 50% chance of having a clinically important disturbance.

- *Satisfaction with care *will be measured with VAS scale ranging from 0 to 10.

- *Patient's assessment of global perceived effect *will be measured by self-assessment on a 7-point scale [32].

### Other variables

The main independent variable is the intervention arm: biopsychosocial multidisciplinary group intervention, or usual clinical care.

The following socio-demographic variables will be recorded: age; sex; educational level (illiterate or non-completed primary studies, completed primary studies, second degree studies, university studies); working situation (housewife, self-employed, employed elsewhere); profession; number of children or other relatives to care for; marital status. Clinical variables: weight and height; previous non-specific acute or sub-acute LBP episodes (none, one or two, three or more); pre-study duration of the current episode of LBP; pain irradiation to the leg (yes or no); severity of pain irradiated to the leg (analogical visual scale); stretching radicular test-Lasegue's maneuver (it has not been carried out, positive if onset of pain irradiated to the leg appears below 30° or between 30-60°, is negative if this pain appears upon 60° or does not appear); prescribed pharmacological treatment: muscle relaxants, analgesics, AINEs, corticoids, ansiolitic drugs, gastric protectors, prescribed diagnostic tests: radiography, Nuclear Magnetic Resonance, Scanner; patient compliance of recommendations and treatments; referrals to other departments (Orthopaedics, Rehabilitation, Neurosurgery, other); Primary Health Care and Hospital emergency visits due to current sub-acute LBP episode; non pharmacological therapeutic measure (relaxation, physiotherapy, reflexology, psychotherapist, etc); vigorous physical activity [33].

### Data collection and follow-up

All the outcomes will be measured at the individual level. All participants will be invited to attend the PHCC for outcome assessments. They will be assessed on his/her first visit to the PHCC, and at 3 months, 6 months, and 12 months after the onset of LBP. The timing for the second assessment was chosen because it would detect the patients' situation near the point at which they would become chronic. The timing for the last assessment was chosen because adequate follow-up time has been recommended by many authors[6,13]

At each assessment, an expert trained fellow (a psychologist) will make up to three phone calls at different times during the day to make the appointments. During the visit, he/she will fill out the questionnaires by interviewing the participant, will do Lasegue's maneuvre test, will collect information by reviewing medical records, will contact the patient's GP to inquire about their development (compliance of treatment, sick leave, evolution to chronic back pain) and will resolve any questions about the conduct of research.

On January 2009, a pilot study was carried out. Its completion with 14 participants allowed us to: discuss the inclusion and exclusion criteria and the questionnaires used (length, understanding and acceptance): plan the recruitment of patients to achieve a suitable number of participants per group (5 out of 7 invited to participate, attended); redefine the length and distribution of the contents of the intervention and assess adherence to the intervention.

The recruitment period is six months.

**Figure 1:**
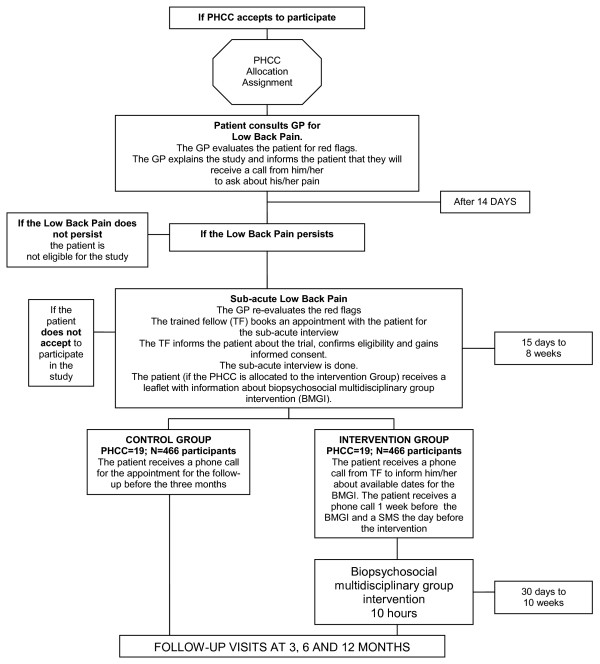
**Study flow chart**. Notes: PHCC = Primary Health Care Centres; GP = General practitioner.

Table 4 gives an overview of data collection. Data were entered in a centralised database by the expert trained fellow and the quality of the data will be revised. Figure 1 shows the trial flow chart of the study.

**Table 4:** Overview of data collection

	*Baseline*	*Follow-up*		
**Outcome Measures**	**15 days after onset of LBP**	**3 months**	**6 months**	**12 months**

Disability: Roland Morris Questionnaire (RDQ)	X	X	X	X

McGill Pain Questionnaire	X	X	X	X

Quality of life Questionnaire (SF-12v2)	X	X	X	X

Duration of the current episode LBP	X	X	X	X

Work sick leave (yes or no)	X	X	X	X

Duration of work sick leave in days	X	X	X	X

Percentage of change in pharmacological treatments	X	X	X	X

Fear Avoidance Beliefs Questionnaire (FAB)	X	X		X

Goldberg Scale (Anxiety and Depression) Questionnaire	X	X		X

Satisfaction with care	X	X	X	X

Patients' perceived global assessment	X	X	X	X

***Independent Variables***				

***Socio-demographic***				

Age, sex, educational level, work situation, profession, number of children, marital status	X			

***Clinical***				

Weight and height	X			X

Previous LBP episodes	X			

Pre-study duration of the current episode of LBP	X			

Pain irradiation to the leg	X			

Severity of the pain irradiated to the leg (VAS)	X			

Stretching radicular test-Lasegue's Maneuver	X			

Prescribed pharmacological treatment	X	X	X	X

Prescribed diagnostic tests: Rx, NMR, Scanner	X	X	X	X

Patient compliance of recommendations-treatment	X	X	X	X

Referrals to other services	X	X	X	X

Primary Health Care and Hospitals emergency visits	X	X	X	X

Non pharmacological therapeutic measures*	X	X	X	X

Vigorous Physical Activity Questionnaire	X	X	X	X

Notes: LBP = Low back pain; VAS = Visual Analog Scale; Rx = Radiography; NMR = Nuclear Magnetic Resonance

* Non pharmacological therapeutic measures such as relaxation, physiotherapy, reflexology, psychotherapist or others

### Sample size

The sample-size calculation is based on change in RDQ at three months after onset of LBP. It is recommended that a change in 2 to 3 points on the RDQ should be considered the minimum clinically important change [22]. To allow for the cluster randomisation by PHCC, we assume an intraclass correlation coefficient of 0.1 [34] and a minimum average number of individuals sampled per PHCC of 25. In order to detect a difference of 2.5 points between the two intervention arms with a standard deviation of 5.7 [22,35], an alpha error of 0.05, a beta error of 0.10, and a 20% dropout rate, a sample size of 932 subjects was required, 466 subjects per intervention arm. Therefore, the total number of PHCCs is 38 (19 for group).

### Statistical Analysis

Data will be analysed in accordance with CONSORT guidelines, extension to cluster randomised trial. Data will be analysed by intention-to-treat. To address potential biases due to incomplete follow-up, we will analyse patients with complete data at all time points and those with data at any time point, using the last known value carried forward to replace missing values. Bias due to non-response will be assessed at each follow-up.

The primary end points for the trial will be 3 months (short term), 6 months and 12 months (long term).

We will obtain descriptive statistics for the outcome measures, baseline characteristics and clinical measures as mean (standard deviation) or numbers (percentages), with 95% confidence intervals at each assessment for all the patients and for each group.

The distribution of data will be checked to ensure that parametric assumptions are met, and non-parametric analyses will be used when appropriate. We will use independent *t *tests or Mann-Whitney U tests for continuous data and Χ^2 ^tests or Fisher's exact probability test for categorical data for unadjusted comparisons between groups at each assessment.

Continuous data for change in scores from baseline will be compared between groups and for each group. The paired t-test for difference will be used to compare the change between pre- and post- intervention.

To decide on an important change in individuals, we will contrast the distributions of changes in all the questionnaires and in each assessment, in individuals who change to chronic state and in individuals who do not [22]. We will do the same between those individuals who return to work and those who do not. We will compare individual change scores to the standard error of measurement (SEM).

To detect changes in groups, the intervention effect will be calculated through the effect-size for each questionnaire and at each assessment. Effect-size was calculated following the method of Kazis et al [36]

We will evaluate responsiveness of each scale by standardised response mean (SRM) [37] and receiver-operating characteristic method (ROC curve). The SRM will be performed between two-time points (baseline and each assessment) and for each intervention arm. We have chosen recovery according to the patient as an external criterion for clinically relevant improvement.

Simple correlations between the scores of the different scales (disability, severity of pain, quality of life and psychosocial scales) will be obtained through the Pearson linear correlation coefficient or the Spearman correlation, in the case of parametric assumptions not being met, at each assessment.

Since the unit of randomisation is the PHCC, we will use a regression analysis of individual level data using methods for clustered data [38]. For adjusting comparisons and to account for cluster randomisation, multilevel linear regression analyses on repeated measures on each of the outcome scales will be used to assess the effect of intervention and to investigate the factors that influence each of the outcome scales at each time point adjusting for baseline scores, baseline characteristics of patients (socio-demographic, clinical and professional) and the other scale scores if they are found to affect outcomes significantly over time. The possible association between intervention and time will be studied. The PHCC and the individual will be considered as random effects and intervention and time as fixed effects.

A multilevel Cox regression model will be used to evaluate the association between the main independent variable, the prognostic factors and the time of subsiding of the current episode of LBP or return to work at 3 months. The patients for whom the duration of the current episode is more than 3 months or those who do not return to work at 3 months will be censored at that time. Kaplan-Meier survival curves will be used to compare the rate of evolution to chronicity and return to work between both groups. Logrank and Breslow test will be used for testing the equality of survival among groups.

The differences in medication prescription at baseline and at each assessment in the interventions and control groups will be compared.

We will calculate the relative risk reduction (RRR) and number needed to treat (NNT) to facilitate development of clinical guidelines for future management.

We will study the interactions and the collinearity. The collinearity of the maximal models will be evaluated using the criteria proposed by Belsley [39]. The significance level of all models will be set at 5%.

The SPSS statistical package for Windows, version 17 (SPSS Inc., Chicago, IL) and the SAS 9.1.3 for Windows (SAS Institute Inc., Cary, NC, USA), will be used for statistical analysis.

### Ethical aspects

The study will be conducted according to Guidelines of the Helsinki Declaration and of Good Clinical Research Practice. The project/study protocol has been approved by the Ethical and Clinical Research Committee of IDIAP Jordi Gol, Institute of Research in Primary Health Care.

Informed Consent: The information will be provided orally as well as written. Study subjects will have sufficient opportunity to ask questions regarding study details. Informed consent follows the guidelines of the Helsinki Declaration.

Data confidentiality: Confidentiality and anonymity of the data will be ensured according to the law 15/1999 of data confidentiality, both in the implementation phase of the project and in presentations or publications resulting there from. Individual data will be codified to ensure anonymity. Only researchers and monitors will have access to the data.

This trial is registered as Current Controlled Trials ISRCTN21392091

## Discussion

Non-specific LBP is a prevalent disorder and a common reason for patient visit to GPs. For about 6% to 10% of patients, the disease may recur or become chronic and the demand on the health-care system is costly. Early identification of clinical, psychosocial and professional risk factors is important to prevent the progression to chronic LBP. Throughout this study, we hope to provide evidence on the effectiveness of the proposed biopsychosocial multidisciplinary intervention in reducing non-specific LBP episodes and its personal, social and economic impact. If the intervention is effective, it could be applied to PHCCs.

There are some limitations in this study. The participants are persons attending a PHCC. Although this aspect can pose a threat to the external validity of the data, the Spanish National Service is universal and free and more than 70% of the general Spanish population seek attention every year in the PHCC [40], and this percentage rises with a wide follow-up. Moreover, in our study population, patients tend to visit the GP because they feel pain.

Acute, sub-acute and chronic LBP is a very common complaint in PHCC. We have assumed that the usual clinical care is homogeneous among clinicians that will participate in the study, namely, the standardized clinical guidelines of the Catalan Health Institute, "Clinical Practice Guidelines in the Pathology of the Lumbar Spine in Adults", to which most of the GPs taking part in the study will belong. These guidelines will be mentioned as the usual clinical care to follow at the presentation session of the study. Moreover, it is known that professionals who participate in research projects tend to be more motivated and experienced. Therefore, we assume that their medical practice involving the studied condition does not differ from that indicated by the guidelines of the Catalan Health Institute. In any case, the effects of a possible variability in the usual clinical practice will be attenuated by our study design, as it will be provided to both groups.

As other studies have found a higher rate and lack of compliance, another possible limitation will be the dropout of follow-up. In the present study, the dropout rate was estimated at 20%, since the recruitment and follow-up of the patients is done by their GPs, and patients tend to visit them when they feel pain. However, we will perform recall mechanisms, and we hope that these strategies will facilitate follow-up and compliance.

It is important to point out some strengths in the research. The literature on risk factors for chronic LBP is abundant with numerous prospective studies done with relatively small samples and assessing only a specific category of chronic LBP risk factors. In the present study, we will recruit a large sample size (n = 932), with a short and long term follow-up assessment and focused on achieving the adherence of the preventive interventions as many authors have emphasized [13]. Moreover, the five domains for clinical research that should be used in all LBP studies (symptoms, function, general well-being, work disability, and satisfaction with care) will be considered [41].

Another strength is the variety of the risk factors addressed (demographic, clinical, psychological, social and professional characteristics), which enables an analysis of interrelations and allows identification and/or confirms the risk factors for chronic LBP.

Furthermore, the sample size is calculated taking into account the intraclass correlation coefficient and the design effect. Cluster specific methods and multilevel analyses will be used, because the PHCC rather than the patients were randomised. Because responsiveness is not an immutable characteristic, but may vary according to population and context [42], each questionnaire's responsiveness will be evaluated in our setting.

Finally, one notable aspect of the biopsychosocial multidisciplinary intervention is the promotion of the transdisciplinarity [43] between professionals, where team members feel empowered by the contribution of other colleagues.

## Competing interests

The authors declare that they have no competing interests. The study sponsors have no role in the study design, the collection, analysis, or interpretation of the data, the writing of the report, or the decision to submit the paper for publication.

## Authors' contributions

All authors were responsible for the conception of the project and drafting the first study proposal. TRB, IFSM, MBC, AB and EPR were involved in writing the manuscript, and all authors critically revised and approved the final manuscript. TRB, IFSM, MBC, JM, ENJ and EPR designed the methodology and TRB designed the statistical analysis. IFSM, MJGM, RMP, MRO and EPR contributed to the description of the background, designed the questionnaires and made the presentation for the recruitment of the PHCC. TRB, MBC, JM, EMM, ENJ, MJGM, MPO, RMP, MNJ and EPR designed the biopsychosocial multidisciplinary intervention and elaborated the educational DVD. AB is the trained fellow and designed the questionnaires and the centralized database. AB, JM, EMM, MJGM and MPO performed the pilot study.

## Pre-publication history

The pre-publication history for this paper can be accessed here:

http://www.biomedcentral.com/1472-6963/10/12/prepub
